# NRICM101 in combatting COVID-19 induced brain fog: Neuroprotective effects and neurovascular integrity preservation in hACE2 mice

**DOI:** 10.1016/j.jtcme.2024.07.001

**Published:** 2024-07-03

**Authors:** Cher-Chia Chang, Yea-Hwey Wang, Jiin-Cherng Yen, Chia-Ching Liaw, Keng-Chang Tsai, Wen-Chi Wei, Wen-Fei Chiou, Chun-Tang Chiou, Kuo-Tong Liou, Yuh-Chiang Shen, Yi-Chang Su

**Affiliations:** a National Research Institute of Chinese Medicine, Ministry of Health and Welfare, Taipei City, 112026, Taiwan; b Institute of Pharmacology, School of Medicine, National Yang Ming Chiao Tung University, Taipei City, 112304, Taiwan; c National Taipei University of Nursing and Health Science, Taipei City, 112303, Taiwan; d Department of Medicine, Mackay Medical College, New Taipei City, 25245, Taiwan; e Department of Chinese Medicine, Tri-Service General Hospital, National Defense Medical Center, Taipei City, 114202, Taiwan; f Graduate Institute of Natural Products, College of Pharmacy, Kaohsiung Medical University, Kaohsiung, 807378, Taiwan; g Program in Medical Biotechnology, College of Medical Science and Technology, Taipei Medical University, Taipei City, 110301, Taiwan

**Keywords:** COVID-19, NRICM101, Traditional Chinese medicine, Brain fog, Neurovasculature disruption, Complement &NET activation

## Abstract

Amidst growing concerns over COVID-19 aftereffects like fatigue and cognitive issues, NRICM101, a traditional Chinese medicine, has shown promise. Used by over 2 million people globally, it notably reduces hospitalizations and intubations in COVID-19 patients. To explore whether NRICM101 could combat COVID-19 brain fog, we tested NRICM101 on hACE2 transgenic mice administered the S1 protein of SARS-CoV-2, aiming to mitigate S1-induced cognitive issues by measuring animal behaviors, immunohistochemistry (IHC) staining, and next-generation sequencing (NGS) analysis. The study revealed that S1 protein-administered mice displayed marked signs of brain fog, characterized by reduced learning, memory, and nesting abilities. However, NRICM101 treatment in these animals ameliorated all these cognitive functions. S1 protein administration in mice induced notable inflammation, leading to the death of neurons (NeuN^+^) and neural stem cells (DCX^+^) in hACE2 transgenic mice. This was accompanied by heightened microglia activation (IBA1^+^/CD68^+^), increased cytokine production (IL1β, IL6), induction of neutrophil extracellular traps (NET), inflammation (NLRP3, CD11b), and platelet (CD31, vWF) and complement (C3) activation, ultimately damaging neurovasculature and disrupting the blood-brain barrier (B.B.B.). Administration of NRICM101 effectively alleviated all these pathological changes. In conclusion, NRICM101 has the potential to prevent COVID-19-associated brain fog by bolstering neurovascular integrity and protecting neurons and neural stem cells. This is achieved by the inhibition of S1 protein-induced complement activation, which in turn leads to the prevention of damage to the neurovasculature and the subsequent death of neurons.

## Graphical abstract

**Figure:**
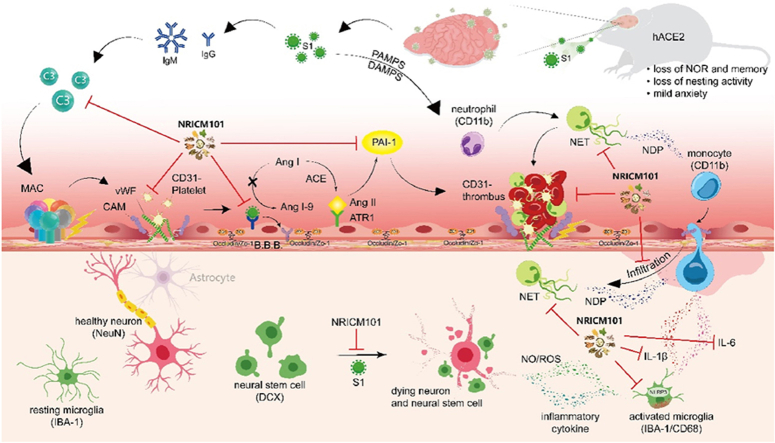

## 1 Introduction

The COVID-19 pandemic has had a profound impact on public health, with initial efforts prioritizing the minimization of mortality risks. However, concern is growing about the enduring effects of long COVID. Long COVID, or post-acute sequelae of COVID-19, refers to persistent symptoms post-recovery. These include fatigue, shortness of breath, chest and joint pain, and cognitive impairment known as "brain fog".^1^, ^2^, ^3^ Brain fog affects memory, attention, and daily function, hampering work and study.^4^ About 10 % of COVID-19 cases lead to long COVID, which potentially affects over 65 million individuals globally.^5^, ^6^, ^7^ This estimate is conservative, excluding asymptomatic cases. The complexity of long COVID underscores the need for research into its causes, risks, and treatments. The mechanisms of brain fog, which are potentially linked to virus-induced neuroinflammation and neuronal cell death,^8^, ^9^, ^10^, ^11^, ^12^ necessitate urgent research to develop effective solutions.

NRICM101, an innovative medication from Traditional Chinese Medicine (TCM), has been administered to over 2 million COVID-19 patients worldwide. The National Research Institute of Chinese Medicine and the National Union of Chinese Medical Doctors' Associations established the NRICM101 Medical Information Platform to support telemedicine for COVID-19 patients. The "NRICM101 Dynamic Inquiry Table" allows users to find medical institutions offering NRICM101 and check daily inventories. Usage statistics are monitored via a system tracking authorized GMP manufacturers and clinic supplies, including data from Taiwan's National Health Insurance Database. Global sales estimates through Amazon suggest over 2.0 million users (https://www.nricm.edu.tw/p/406-1000-6773,r61.php?Lang=zh-tw). In mid-2021, during the alpha variant dominance, hospitalized COVID-19 patients in Taiwan received NRICM101.^13^ Our study (840 subjects included) assessed the link between NRICM101 use and intubation/ICU admission in patients not needing oxygen support. After matching (149 pairs), patients taking NRICM101 showed no intubation/ICU cases.^14^ Our bedside-to-bench study suggests that NRICM101 may disrupt disease progression through its antiviral and anti-inflammatory properties, offering promise as a multi-target agent for the prevention and treatment of COVID-19.^15^, ^16^, ^17^, ^18^ NRICM101 effectively protects against SARS-CoV-2-S1-induced pulmonary injury via modulation of the innate immune response, pattern-recognition receptor, and toll-like receptor signaling pathways to ameliorate diffuse alveolar damage (*DAD*).^13^^,^^19^^,^^20^ Further protective effects of NRICM101 and its enhanced version (NRICM102) have been extensively reported.^21^^,^^22^ This research aims to further explore effectiveness and mechanism of action of NRICM101 in averting COVID-19-induced brain fog. We employed hACE2 transgenic mice administered the S1 subunit of the SARS-CoV-2 spike protein for this investigation.

## 2 Materials and methods

### 2.1 Preparation of NRICM101 decoction

The NRICM101 decoction, consisting of 10 traditional Chinese medicines (TCMs) produced according to our previous reports,^13^^,^^20^ was prepared by Dr. Chia-Ching Liaw, one of the coauthors and curator of the Herbarium of the National Research Institute of Chinese Medicine (NRICM, Taipei, Taiwan). It consisted of 10 herbs: Scutellaria Root (*Scutellaria baicalensis*, HA, 18.75 g), Heartleaf Houttuynia (*Houttuynia cordata*, HC, 18.75 g), Mulberry Leaf (*Morus alba*, NB, 11.25 g), Saposhnikovia Root (*Saposhnikovia divaricata,* NC, 7.50 g), Mongolian Snakegourd Fruit (*Trichosanthes kirilowii*, ND, 18.75 g), Indigowoad Root *(Isatis indigotica,* NE, 18.75 g), baked Liquorice Root (*Glycyrrhiza glabra*, NG, 7.50 g), Magnolia Bark *(Magnolia officinalis*, NK, 11.25 g), Peppermint Herb (*Mentha haplocalyx,* NL, 11.25 g), and Fineleaf Nepeta (*Nepeta tenuifolia,* NR, 11.25 g).

For quality control, the HPLC fingerprint profiles of the NRICM101 decoction, 10 single herbs, and 12 batches with the 17 active components of NRICM101 have been analyzed as follows: (A). The HPLC profiles of NRICM101 decoction at three different wavelengths: 210, 254, and 280 nm. 1: 3-*O*-Caffeoylquinic acid; 2: Epigoitrin; 3: 5-*O*-Caffeoylquinic acid; 4: 4-*O*-caffeoylquinic acid; 5: Rutin; 6: Chrysin 6-*C*-arabinoside-8-*C*-glucoside; 7: Liquiritin; 8: Acetoside; 9: Quercetin 3-galactoside; 10: Quercetin 3-glucoside; 11: Chrysin 6-*C*-glucoside-8-*C*arabinoside; 12: Scutellarin; 13: Quercetin 3-rhamnoside; 14: Baicalin; 15: Norwogonin 7-*O*-glucuronide; 16: Oroxyloside; 17: Wogonoside. (B). The HPLC fingerprints of the 10 single herbs at 280 nm. HA: Scutellaria root (*Scutellaria baicalensis*); HC: Heartleaf Houttuynia (*Houttuynia cordata*); NB: Mulberry Leaf (*Morus alba*), NC: Saposhnikovia Root (*Saposhnikovia divaricata*); ND: Mongolian Snakegourd Fruit (*Trichosanthes kirilowii*); NE: Indigowoad Root (*Isatis indigotica*); NG: honey-fired Liquorice Root (*Glycyrrhiza glabra*); NK: Magnolia Bark (Magnolia officinalis); NL: Peppermint Herb (*Mentha haplocalyx*); NR: Fineleaf Schizonepeta Spike (*Schizonepeta tenuifolia*). (C). The HPLC fingerprints of 12 batches of decoction obtained from the TCM pharmacies of two medical centers at 280 nm. (Supporting Fig. 1;^13^).

**Fig. 1:**
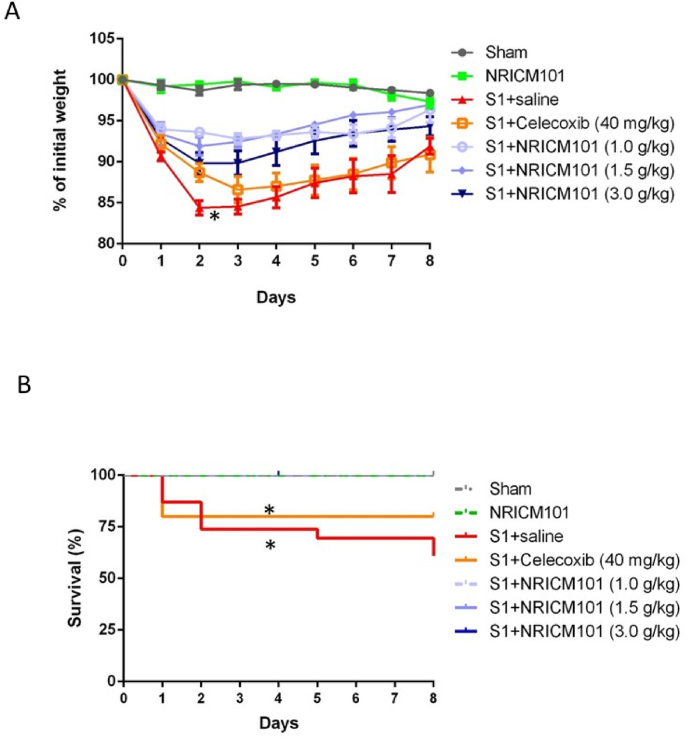
Effects of NRICM101 on changes in (A) body weight and (B) survival rates after SARS-CoV-2 spike protein S1 administration. The experimental protocol was designed and performed on hACE2 mice with SARS-Co V-2-S1 protein (400 μg/kg)-induced brain fog divided into 4 subgroups, including (1) saline control (Sham), (2) S1 plus saline-treated (S1+saline), (3) S1 plus NRICM101 (1.0 g/kg, p.o.)-treated, (4) S1 plus NRICM101 (1.5 g/kg, p.o.)-treated, (5) S1 plus NRICM101 (3.0 g/kg, p.o.)-treated, (6) S1 plus celecoxib (40 mg/kg, p.o.)-treated, and (7) NRICM101 (1.5 g/kg, p.o.)-treated only (NRICM101) group. NRICM101 was administered orally once daily for eight consecutive days, starting 1 day after S1 protein administration (day 0) and continuing for 8 consecutive days post-S1 administration. The body weights and survival rates of mice were carefully observed and recorded. Using one-way ANOVA and subsequent Newman–Keuls post hoc test for multiple comparisons against the sham group, *p was found to be < 0.05 (n = 10 for each group).

### 2.2 Animal grouping and administration of S1 protein in hACE2 mouse model

Male hACE2 transgenic mice (The Jackson Laboratory, USA) weighing 28–30 g were used in this experiment, and the protocol was reviewed and approved by the Animal Research Committee of the National Research Institute of Chinese Medicine (approval number: NRICM-IACUC-111-912-3). All mice were fed in controlled conditions (12 h light/dark cycle with humidity of 60–70 %, 21–23 °C) with free access to food and water. Mice were randomly assigned into 4 groups (n = 10 for each group): (1) a sham group (Sham), (2) NRICM101 group, (3) S1 protein plus saline group, (4) S1 protein plus NRICM101 (1.0, 1.5, 3.0 g/kg) group, and S1 protein plus a reference drug celecoxib (40 mg/kg) group.^23^ For S1 protein administration in the hACE2 mouse model, mice were anesthetized with a mixture of 1.5–2% isoflurane and oxygen. For brain fog induction, SARS-CoV-2-S1 protein (400 μg/kg, P.O.) in 30 μl saline was microinjected carefully into the internal carotid artery of hACE2 mice. NRICM101 was administered orally once daily for eight consecutive days, starting 1 day after S1 protein administration (day 0) and continuing for 8 consecutive days post-S1 administration. Mice were monitored daily for body weight changes for 8 consecutive days. All the following behavioral tests were performed on the 7th or 8th day.

### 2.3 Assessment of neurological deficit and analysis of survival rate

The neurological function of the mice was assessed by analyzing their tracking distance and tracks within 5 min in a behavioral observation box (60 x 40 × 60 cm^3^) before and after S1 protein administration. The results were then analyzed using a video-tracking system (SMART v2.5.21, Panlab, Spain). Survival rates were calculated from day 0 (administration of S1 protein) to day 8.

### 2.4 Novel object recognition test

The novel object recognition test was similar to those in previous studies.^24^^,^^25^ The test included three sessions: training (memory acquisition) (on 7th day), delay (memory consolidation) and test (memory expression) sessions (on 8th day). Before training, mice were individually habituated to a 40 x 60 x 40 behavior observation box for 5 min and the track of the animal was recorded by camera. After 5 min, the animals were removed from the box and placed back into their home cage, and the box was cleaned with a 70 % ethanol solution. In the training trial, mice were placed in an arena containing two identical objects for 10 min. The two identical objects were placed in the box for the animals to explore freely, and a mouse was considered to be exploring an object when its nose was within 2 cm of the object. If the animals explored the two objects for less than 10 s during the 10-min period, the mice were excluded from further experiments. The tested mice were then returned to the home cage for memory consolidation (delayed). After 24 h, one of the two identical objects in the training phase was replaced with a new object. Then the mice were moved from the home cage to the observation box for 10 min, and the time spent exploring the familiar and novel objects was calculated using a video-tracking system (SMART v2.5.21, Panlab, Spain). Recognition memory was scored using the recognition index for each mouse with formula (N or F)/(N + F)%. The recognition index represents the difference between the time spent exploring the novel (N) and familiar (F) objects and the total time spent exploring both objects. Typically, test mice will spend more time on the novel object.

### 2.5 Rotarod test

The rotarod test has been used to assess motor coordination and motor learning as well as endurance. The mice were placed on a rotarod with a diameter of 3 cm. This rod was divided into 6 tracks with a width of 6 cm facing away from the experimenter, and the rotation speed of the rod was increased from 0 rpm to 20 rpm (RPM20) or 50 rpm (RPM50) for an endurance or a coordination test, respectively. After the last mouse fell from the rod, or if 300 s elapsed, the mice were given a 5-min rest and then placed back on the rod for the next trial. Five trials were conducted.^26^

### 2.6 Nesting test

The nesting test was similar to that used in previous studies.^27^, ^28^, ^29^ Two nestlets (approximately 5 g) were placed into the home cage 1 h before the dark cycle (6th day), and then the nest scores were determined after 48 h (8th day). The nest was scored using a 0–5 rating scale, where a score of 0 indicated nestlets not touched, with no shreds torn from the nestlets; 1, nestlets slightly touched, but more than 80 % intact with some shreds picked out; 2, nestlets obviously touched, with many shreds picked out around the nestlets; 3, recognizable nest with a small hollow in the center and minor wall construction; 4, nest with an obvious hollow in the center and walls higher than mice; and 5, perfect bowl-shaped nest with walls higher than mice.

### 2.7 Immunohistochemistry

Fifteen to twenty serial sections (30 μm) were collected from the same parts of the brains of mice from all treatment groups for immunohistochemical analysis. Before staining with specific antibodies, we prepared the tissue sections using the general protocol featuring fixation, permeabilization, and blocking. We then randomly selected tissue slices for overnight incubation at 4 °C in phosphate-buffered saline (PBS) containing 3 % albumin and stained them for specific protein markers using primary antibodies as follows: SARS-CoV-2 spike protein subunit 1 (S1) RBD (1:100), CCL11 (1:50), *citrullinated* histone *H3* (CitH3, NET, 1:50), MPO (1:100), IL1β (1:100), IL-6 (1:100), vWF (1:100), PAI-1 (1:100), NeuN (1:500) and Occludin (1:100), all obtained from GeneTex (Irvine, CA, USA); IBA1 (1:100) and ZO-1 (1:200) from iREAL (Taipei, Taiwan); CD68 (1:50) obtained from BD (San Diego, CA, USA); NLRP3 (1:100) obtained from Cell Signaling Technology Inc. (MA, USA); and C3 (1:50), CD11b (1:50) and CD31 (also known as platelet endothelial cell adhesion molecule 1 [PECAM-1]) (1:50) and doublecortin (DCX, 1:1000) obtained from Abcam (Cambridge, UK). We washed the tissue sections thoroughly and then stained them with secondary antibodies conjugated with Alexa Fluor 488, 555, or 647 (Cell Signaling Technology, Danvers, MA, USA). We mounted the correctly stained sections on coverslips in medium containing 4′,6-diamidino-2-phenylindole (DAPI) and imaged them using a confocal Zeiss LSM780 laser-scanning microscope (Carl Zeiss, Jena, Germany). Using Zen 2011 (black edition, Carl Zeiss MicroImaging, 1997–2011) and AlphaEase FC (Alpha Innotech, San Leandro, CA, USA), we identified, counted, and calculated the area covered by the immunopositive cells or identified the immunopositive proportion of the total area they comprised (as a percentage). We applied this procedure to the whole field of view in regions of interest that we sampled from each group. We used 30–100 × magnification and performed 3–5 independent replications of each experiment. The areas with positive fluorescence staining were determined using the following procedure: Initially, every fluorescence channel was transformed to green fluorescence using Zen 2011, Black Edition by Carl Zeiss. Afterward, the AlphaEaseFC software (version 4.0 by Alpha Innotech) was employed to fine-tune the contrast and sensitivity of the fluorescence region, setting the comprehensive analysis area. This software then computed the proportion of the fluorescent region.

### 2.8 RNA expression

RNA expression was determined by next-generation sequencing (NGS). SimpliNano™ Biochrom Spectrophotometer (Biochrom, MA, USA) was used to check the purity and quantity of each RNA sample prepared from 4 different groups. RNA degradation and integrity were monitored with a Qsep 100 DNA/RNA Analyzer (BiOptic Inc., Taiwan). Sequencing libraries were generated from the total RNA of the samples with a KAPA mRNA HyperPrep Kit (KAPA Biosystems, Roche, Basel, Switzerland). High-throughput sequencing (Illumina NovaSeq 6000 platform) was performed to obtain the raw data, and FastQC and MultiQC were used to assess quality. High-quality raw paired-end read data were obtained by using Trimmomatic (v 0.38) for subsequent analysis. Alignment of read pairs from each sample to a reference genome was performed in HISAT2 software (v 2.1.0), after which the reads mapped to individual genes were counted in FeatureCounts (v 2.0.0). Differentially expressed gene (DEG) analysis of case and control samples was performed with DEGseq (v 1.40.0) or DESeq2 (v 1.26.0). Gene Ontology (GO) functional annotation and Kyoto Encyclopedia of Genes and Genomes (KEGG) pathway enrichment analyses were performed by using clusterProfiler (v 3.14.3). A protein–protein interaction (PPI) network was constructed for the differentially expressed genes using STRINGdb (https://string-db.org/).^20^^,^^22^

### 2.9 Statistical analysis

All results in this study are presented as the mean ± SEM, and the differences between each group were analyzed using one-way analysis of variance (ANOVA) followed by the post hoc Newman–Keuls test for multiple comparisons. Values of p < 0.05 were considered significant. Survival rates was analyzed using Log-Rank test followed by the Holm–Sidak method. Values of p < 0.05 were considered significant.

## 3 Results

### 3.1 The effects of NRICM101 on changes in body weight and survival rate after SARS-CoV-2 spike protein S1 administration

To assess the impact of NRICM101 (1.0, 1.5, 3.0 g/kg) on changes in body weight and survival rate following S1 protein administration, we monitored weight fluctuations and survival rates from day 0 to day 8. Our observations revealed marked decreases in the body weight of mice exposed to S1 protein administration on days 1 and 2, regardless of NRICM101 treatment. This decline was especially notable in the untreated group. However, the body weight gradually returned to a level similar to the baseline over time (Fig. 1A). In terms of survival rate, the group that received S1 protein without treatment exhibited a survival rate of only 60 % by day 8. In contrast, no instances of animal mortality were observed in the group treated with NRICM101 (1.0–3.0 g/kg) (Fig. 1B). However, the group treated with the reference drug celecoxib (40 mg/kg) exhibited a survival rate of 80 % from day 1 to day 8.

### 3.2 The effects of NRICM101 on changes in neurological deficits and anxiety after SARS-CoV-2 spike protein S1 administration

**Fig. 2:**
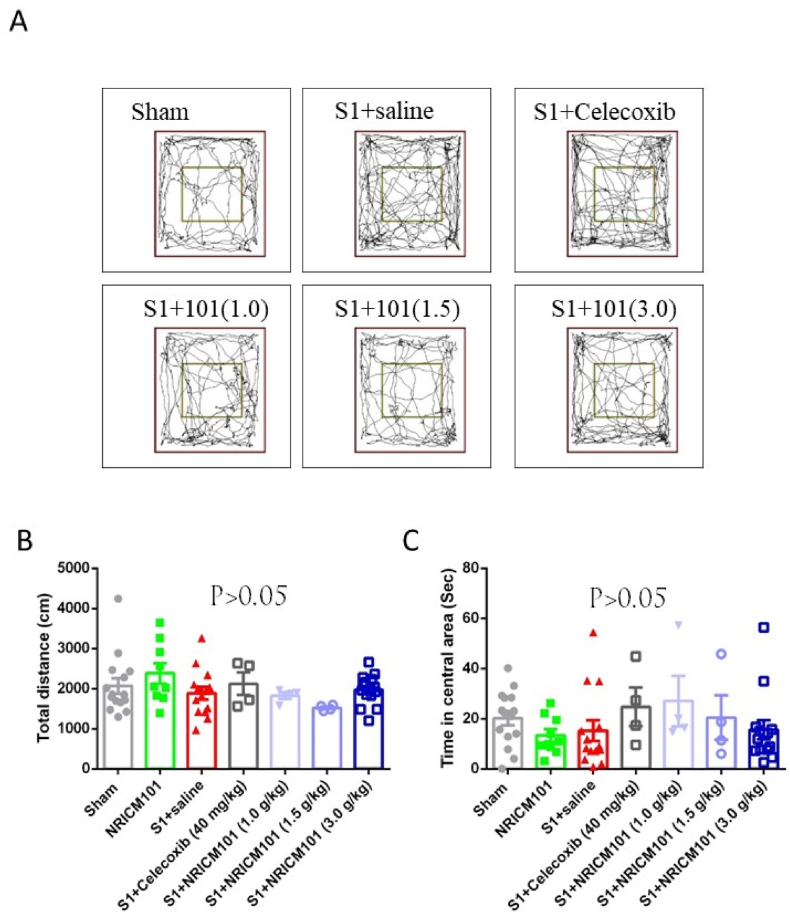
Effects of NRICM101 on changes in (A, B) neurological deficits and (C) anxiety after SARS-CoV-2 spike protein S1 administration. The experimental protocol was designed and performed on hACE2 mice with SARS-CoV-2-S1 protein (400 μg/kg)*-*induced brain fog divided into 7 subgroups as described in Fig. 1. Mice were carefully observed and data was recorded on the 8th day. Using one-way ANOVA and subsequent Newman–Keuls post hoc test for multiple comparisons against the sham group, p was found to be > 0.05 (n = 9–11 for each group).

To evaluate the locomotor activity and assess anxiety-like behavior subsequent to S1 protein administration, we employed an open field test to monitor the movement and paths taken by the animals during a 5-min observation period. Specifically, the total distance covered by the animals provided an indicator of their locomotor activity, while the time spent in the central area of the field was employed to gauge anxiety-like behavior. Animals experiencing depression or anxiety tend to favor corner areas and thus spend less time in the central region. Analysis of locomotor activity revealed that mice across all groups exhibited normal tracking distances (Fig. 2A). Furthermore, there were no notable variations among the groups in terms of a preference for the central area of the field (Fig. 2B). However, the mice in the S1 protein group displayed a lower central residence time than the other groups did. Based on these findings, it is reasonable to hypothesize the presence of mild anxiety subsequent to S1 protein administration (Fig. 2C). However, no statistical significance was found (p > 0.05).

### 3.3 The effects of NRICM101 on changes in novel object recognition and memory after SARS-CoV-2 spike protein S1 administration

**Fig. 3:**
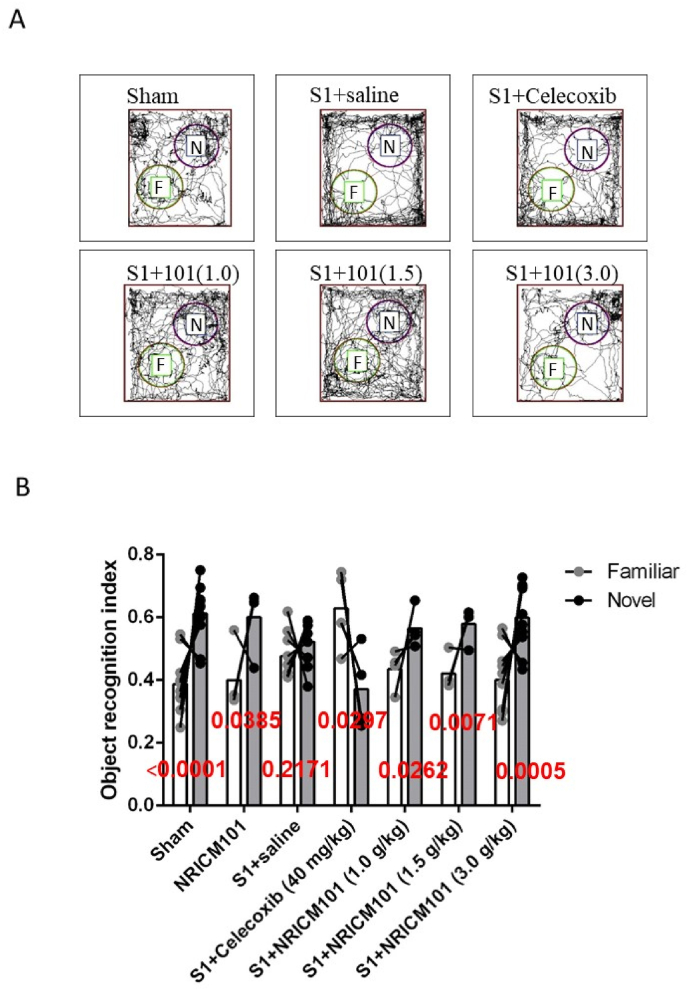
Effect of NRICM101 on changes in novel object recognition memory (NOR) after SARS-CoV-2 spike protein S1 administration. The experimental protocol was designed and performed on hACE2 mice with SARS-Co V-2-S1 protein (400 μg/kg)*-*induced brain fog divided into 7 subgroups as described in Fig. 1. The NOR in mice were carefully recorded (A) on the 8th day and (B) a statistical summary of objective recognition index was calculated and analyzed. A p-value was assigned to each group using a student's t-test (n = 3–6 for each group).

To explore whether NRICM101 treatment mitigated the impairment of novel object recognition memory resulting from S1 protein administration, we employed the novel object recognition test to assess learning memory. Our observations revealed that mice administered the S1 protein spent less time interacting with the novel object compared to the sham, NRICM101-only, and S1 protein + NRICM101 groups (Fig. 3A and B). This finding indicates that S1 protein administration led to a deficiency in novel object recognition and memory (*p* < 0.05). However, treatment with NRICM101, but not the reference drug celecoxib (40 mg/kg), was shown to recover novel object recognition (NOR) and memory to the levels of the normal (sham) group.

### 3.4 The effects of NRICM101 on changes in coordination and endurance after SARS-CoV-2 spike protein S1 administration

**Fig. 4:**
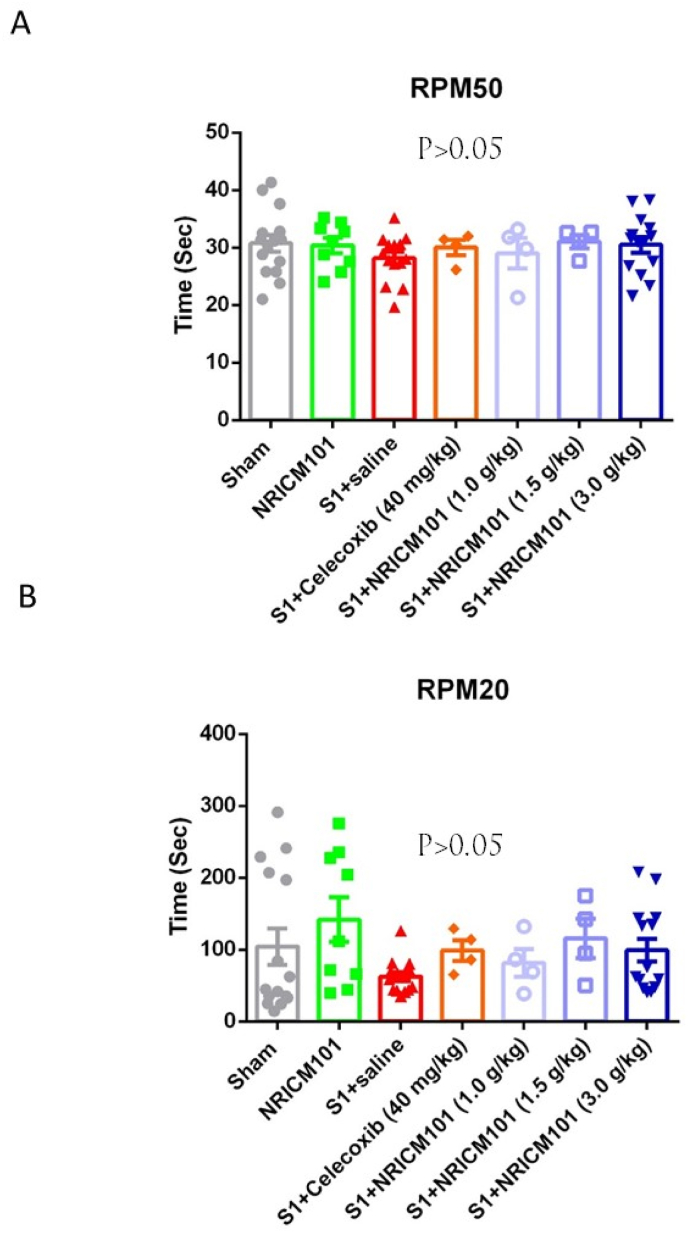
Effects of NRICM101 on changes in (A) coordination and (B) endurance after SARS-CoV-2 spike protein S1 administration. The experimental protocol was designed and performed on hACE2 mice with SARS-Co V-2-S1 protein (400 μg/kg)*-*induced brain fog divided into 7 subgroups as described in Fig. 1. The coordination (RPM50) and endurance (RPM20) in mice were carefully recorded and analyzed on the 8th day. Using one-way ANOVA and subsequent Newman–Keuls post hoc test for multiple comparisons against the sham group, p was found to be > 0.05 (n = 9–11 for each group).

To assess the impact of S1 protein administration on coordination and endurance, we employed a rotarod test. Analysis of the rotarod test outcomes revealed no substantial variations across the different groups (Fig. 4A and B). This outcome suggests that there were no discernible alterations in coordination and endurance following S1 protein administration (p > 0.05).

### 3.5 The effects of NRICM101 on changes in nesting activity after SARS-CoV-2 spike protein S1 administration

**Fig. 5:**
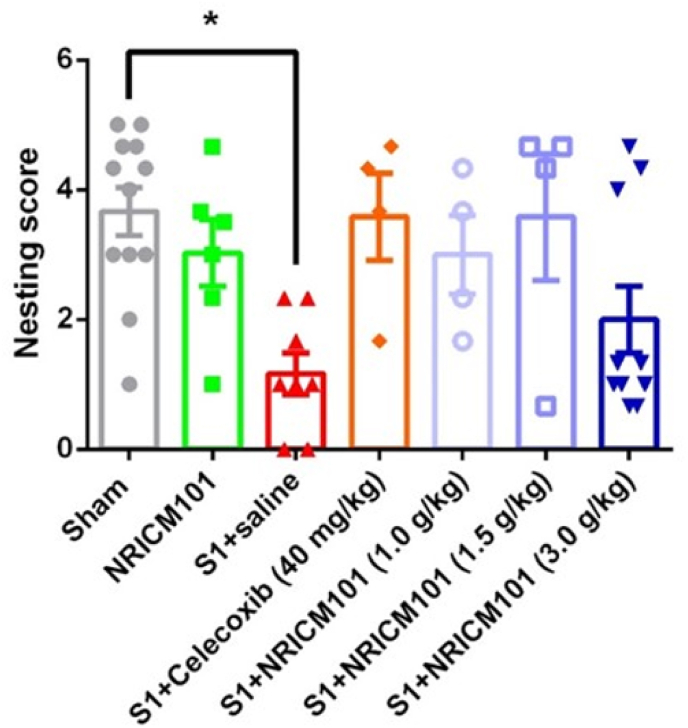
Effect of NRICM101 on changes in nesting activity after SARS-CoV-2 spike protein S1 administration. The experimental protocol was designed and performed on hACE2 mice with SARS-Co V-2-S1 protein (400 μg/kg)*-*induced brain fog divided into 7 subgroups as described in Fig. 1. The nesting activity in mice were carefully recorded (A) on the 8th day and (B) a statistical summary of nesting score was calculated and analyzed. Each comparing group displayed a p-value as indicated, determined using a student's t-test (n = 4–7).

Nesting is an inherent behavior observed in many animals,^30^ and its regular occurrence is indicative of an animal's overall well-being.^31^ To assess nesting behavior, a nesting score was established with six grades. The outcomes revealed that sham mice exhibited an average nesting score of 5 points; this score was mirrored in mice administered only NRICM101. Conversely, mice administered the S1 protein displayed notably lower nesting ability, with an average score of approximately 1 point (Fig. 5A, supplementary). Notably, S1 mice treated with NRICM101 had an average score of 3 points. This score was statistically distinct from that of the group with S1 protein administration (p < 0.05), while not significantly differing from those of the sham and NRICM101-alone groups (Fig. 5). These findings underscore the positive impacts of NRICM101 and the reference drug celecoxib on nesting ability.

### 3.6 The effects of NRICM101 on changes in cerebral microvascular inflammation and neuronal death after SARS-CoV-2 spike protein S1 administration

**Fig. 6:**
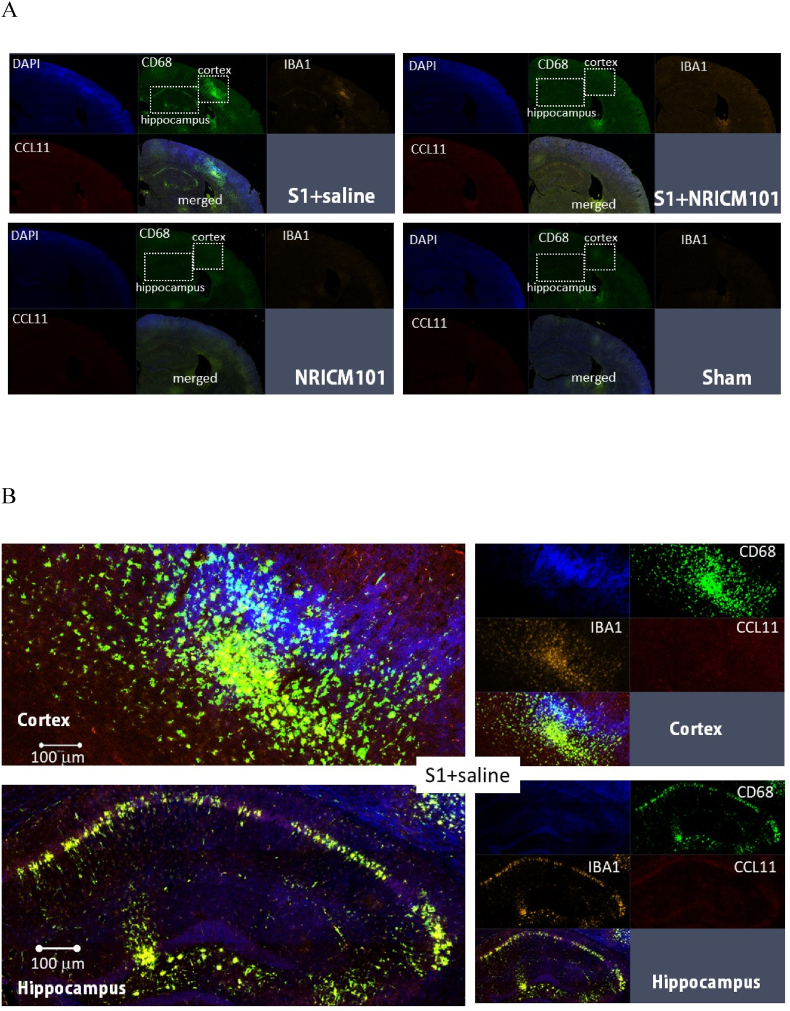

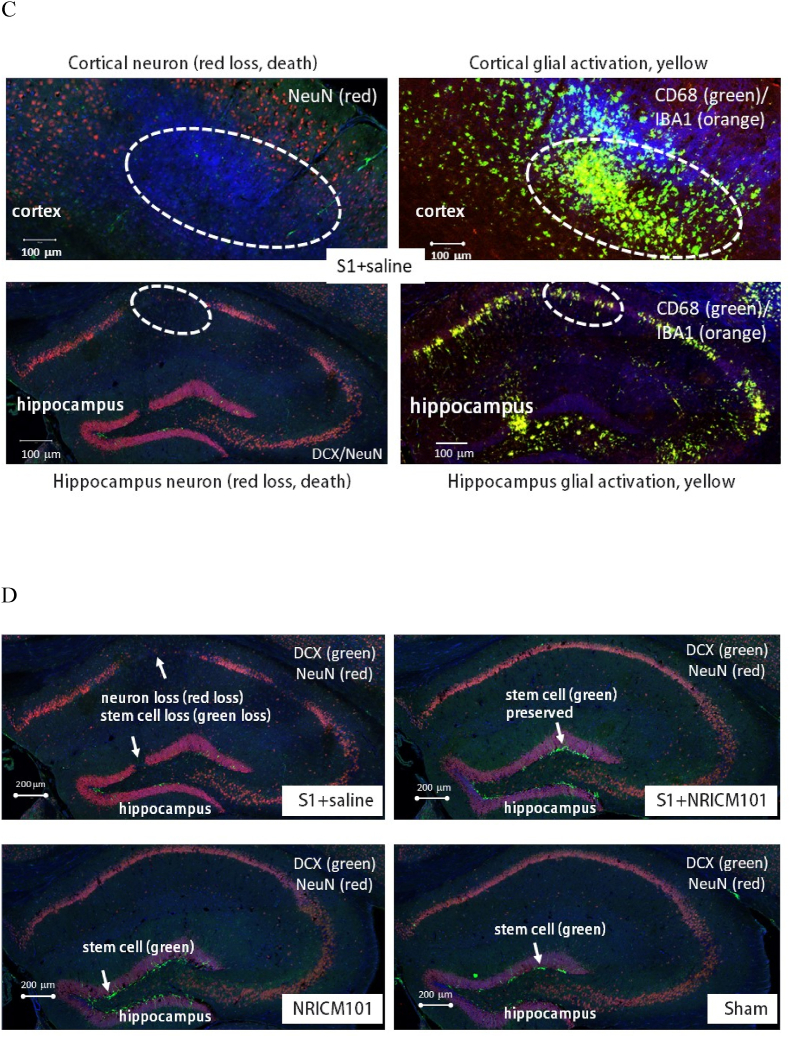
Effects of NRICM101 on changes in cerebral microvascular inflammation and neuronal death after SARS-CoV-2 spike protein S1 administration. The experimental protocol was designed and performed on hACE2 mice with SARS-Co V-2-S1 protein (400 μg/kg)*-*induced brain fog divided into 4 subgroups as described in Fig. 1. The changes in the expression of (A, B) CD68 (green), IBA1 (orange), and CCL11 (red), (C) CD68 (green), IBA1 (orange), and NeuN (red); dashed circle indicates loss of neurons (red, left panel) due to glial cell activation (yellow, right panel), (D) DCX (green) and NeuN (red) within cortex or hippocampus from brain tissue sections were carefully stained and observed with a confocal Zeiss LSM780 laser-scanning microscope on the 8th day; and (H) a statistical summary of all markers was calculated and analyzed. A statistical summary of all markers was calculated and analyzed (Fig. 7H). *p < 0.05 using one-way ANOVA followed by the post hoc Newman–Keuls test for multiple comparisons as compared to S1 + saline group (n = 3–5 for each group) .

**Fig. 7:**
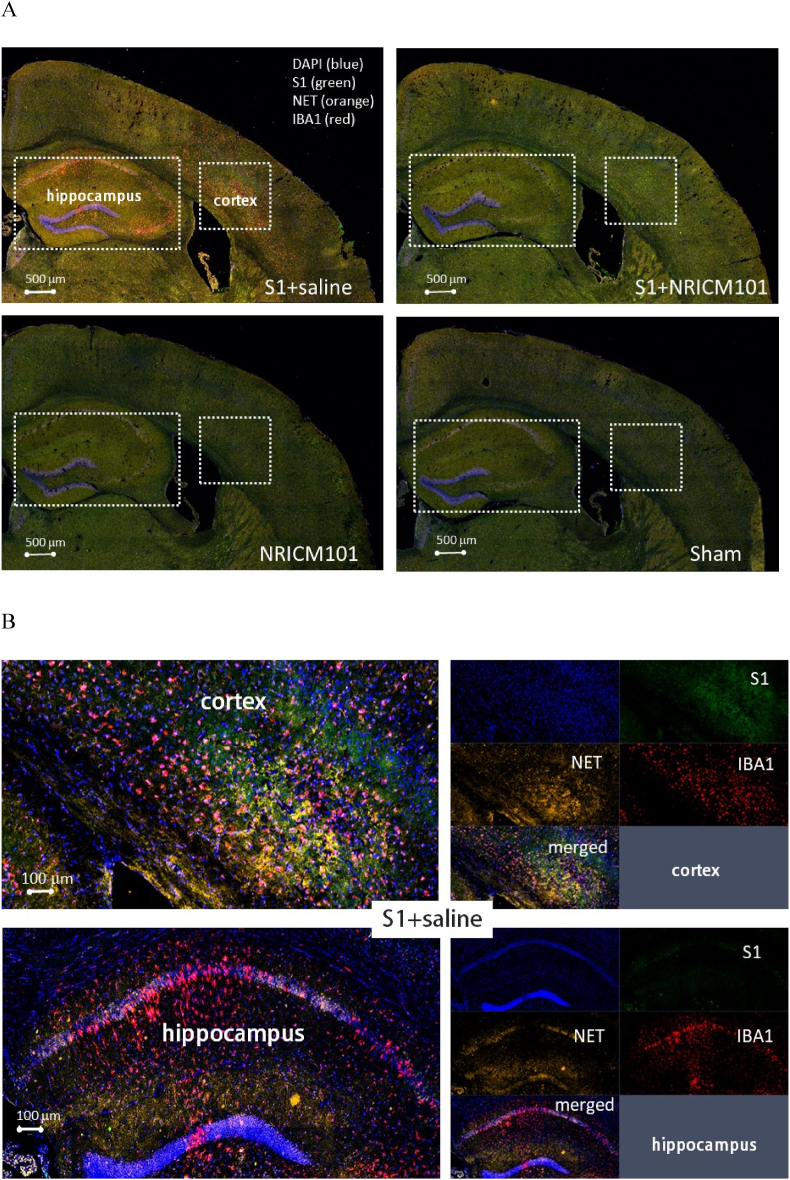

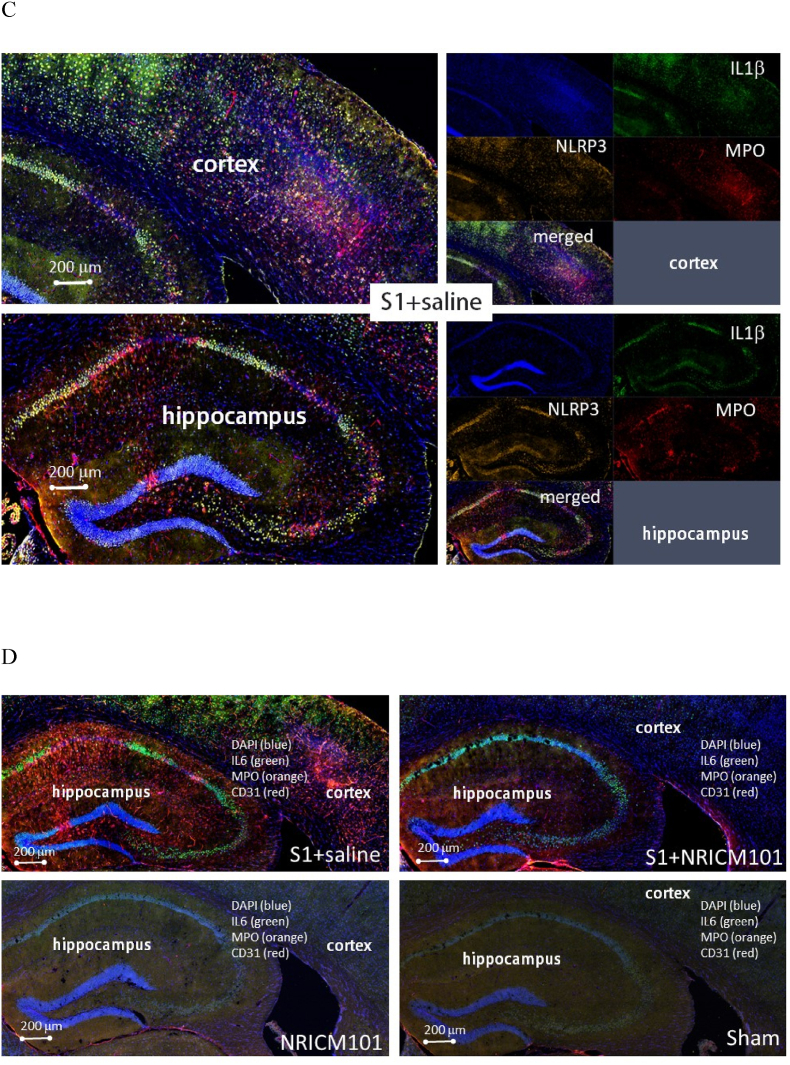

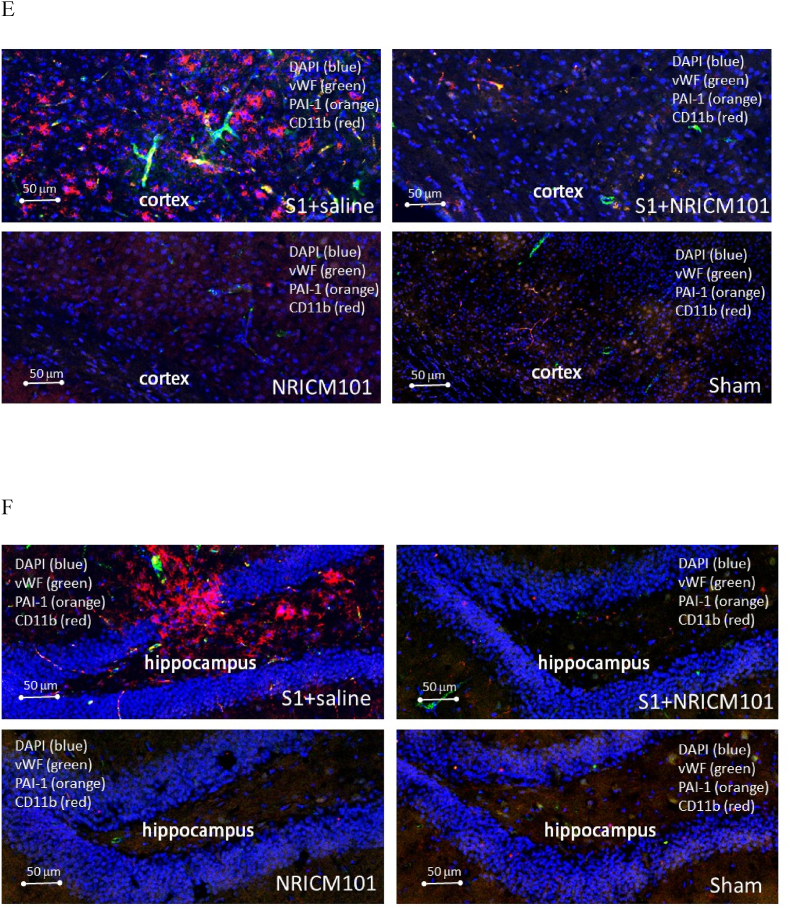

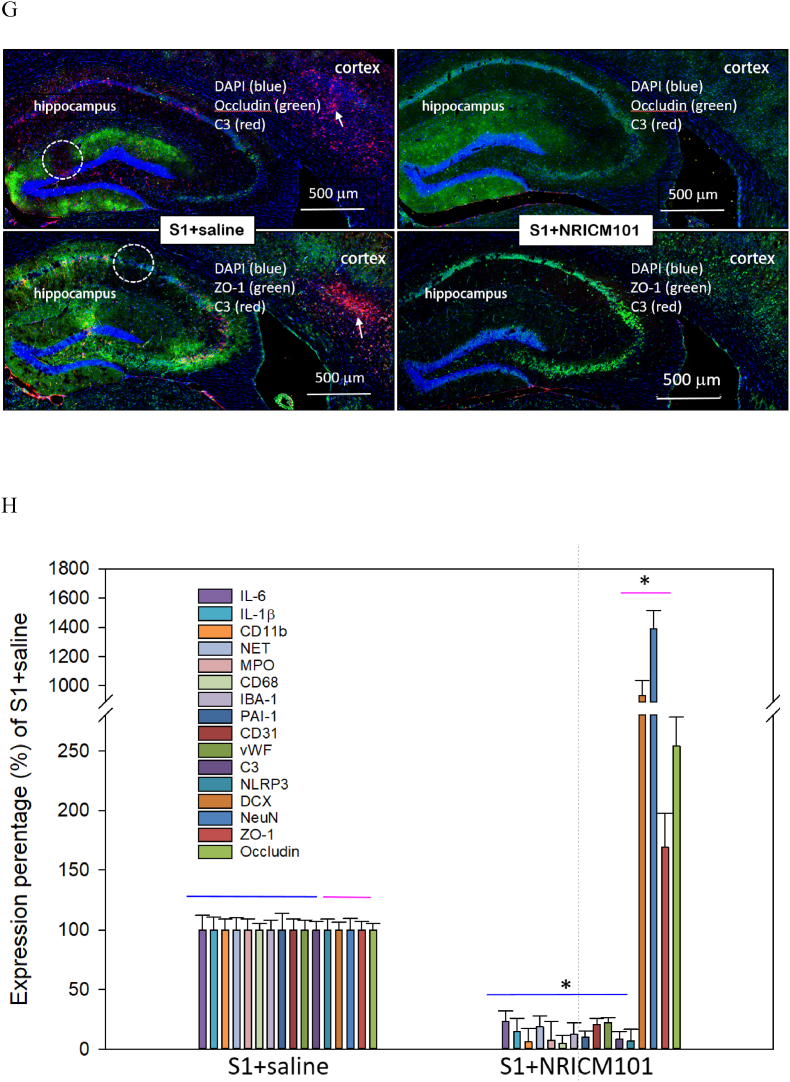
Effects of NRICM101 on changes in cerebral microvascular inflammation and neuronal death after SARS-CoV-2 spike protein S1 administration. The experimental protocol was designed and performed on hACE2 mice with SARS-Co V-2-S1 protein (400 μg/kg)*-*induced brain fog divided into 4 subgroups as described in Fig. 1. The changes in the expression of (A, B) S1 (green), NET (orange), and IBA1 (red), left panel showing co-localization of the 3 stains from right panel, (C) IL1β (green), NLRP3 (orange), and MPO (red), (D) IL6 (green), MPO (orange), and CD31 (red), left panel showing co-localization of the 3 stains from right panel, (E, F) vWF (green), PAI-1 (orange), and CD11b (red), (G) occludin (upper panel, green) or ZO-1 (lower panel, green) and C3 (red, arrows), and dash circles indicate loss of B.B.B. components (green) within cortex or hippocampus from brain tissue sections were carefully stained and observed with a confocal Zeiss LSM780 laser-scanning microscope on 8th day; blue color indicates DAPI staining (DNA). (H) A statistical summary of all markers was calculated and analyzed. *p < 0.05 using one-way ANOVA followed by the post hoc Newman–Keuls test for multiple comparisons as compared to S1 + saline group (n = 3–5 for each group) .

Microglial activation and the production of a specific cytokine, CCL11, are believed to play pivotal roles in the brain fog experienced due to SARS-CoV-2 administration, as reported previously.^10^^,^^11^ To investigate whether brain inflammation and neuronal cell death contributed to the brain fog induced by administration of the S1 protein in this research, we assessed microglial cell activation, inflammation, cytokine production, and neuronal cell death. Our findings revealed robust microglial cell activation (IBA1+/CD68+) in the vicinity of both the cortex and hippocampus (Fig. 6A and B), accompanied by significant losses of mature neurons (NeuN+) and neural stem cells (DCX+) (Fig. 6C and D). Additionally, the activation of microglial cells coincided with the presence of NETs (Fig. 7A and B), a distinct indicator of S1 protein administration,^22^ along with the production of inflammatory markers such as NLRP3, MPO, and IL-6 and IL-1β, rather than CCL11 (Fig. 7C and D). S1 protein administration also initiated the formation of microvascular thrombosis (vWF) through PAI-1-mediated activation of platelets (CD31) and infiltration of neutrophils (CD11b) (Fig. 7E and F).^22^ Importantly, within the regions exhibiting S1-induced damage, significant disruption and permeability of the blood-brain barrier (B.B.B.) were observed (Fig. 7G). This was contrasted by the loss of occludin and ZO-1, two markers of the B.B.B. components, as well as the activation of the complement cascade, as evidenced by the substantial production of C3 (Fig. 7G and H). Notably, all the pathological changes induced by S1 protein administration were markedly attenuated (p < 0.05) by treatment with NRICM101, as demonstrated in Fig. 7, Fig. 9.

**Fig. 8:**
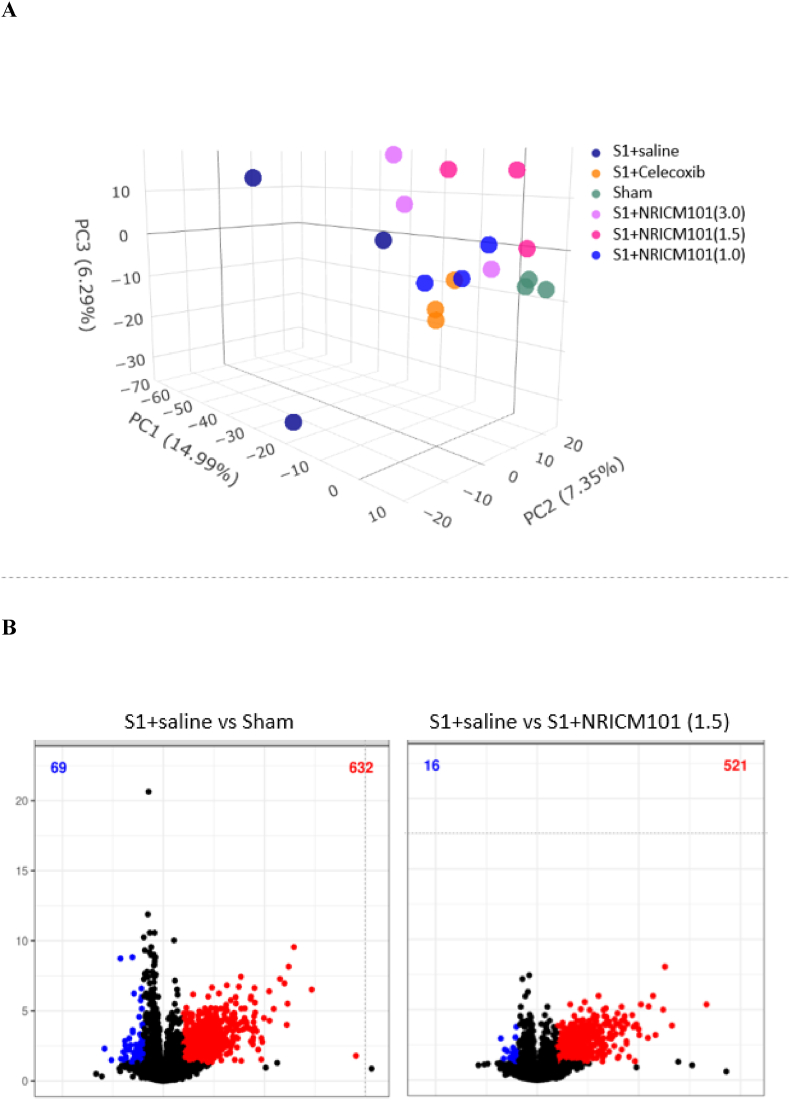
Effects of NRICM101 on changes in gene expression profiling after SARS-CoV-2 spike protein S1 administration. The experimental protocol was designed and performed on hACE2 mice with SARS-Co V-2-S1 protein (400 μg/kg)*-*induced brain fog divided into several subgroups as described in Fig. 1. We assessed changes in gene expression using next-generation sequencing (NGS) and reverse-transcription polymerase chain reaction (RT-PCR) assays to identify differentially expressed genes (DEGs) in the S1+NRICM101 group versus the S1+saline group. (A) a principal component analysis (PCA) plot of the DEGs profiles of 6 treatment groups (n = 3, each group). (B) a volcano plot showing the DEGs numbers (blue, down regulation; red, upregulation) between S1+saline vs. sham (lower left), and S1+NRICM101 (1.5 g/kg) vs. S1+saline (lower right). (C) the disease ontology (DO) gene-concept network was adopted to display and take account the potential biological complexity that a gene may belong to multiple annotation categories and provides change information using DEGs from S1+saline vs. sham groups after S1 treatment. (D) A KEGG (Kyoto Encyclopedia of Genes and Genomes) plot using DEGs from S1+saline vs. sham groups to show the strong activation of complement and coagulant cascade after S1 treatment.

**Fig. 9:**
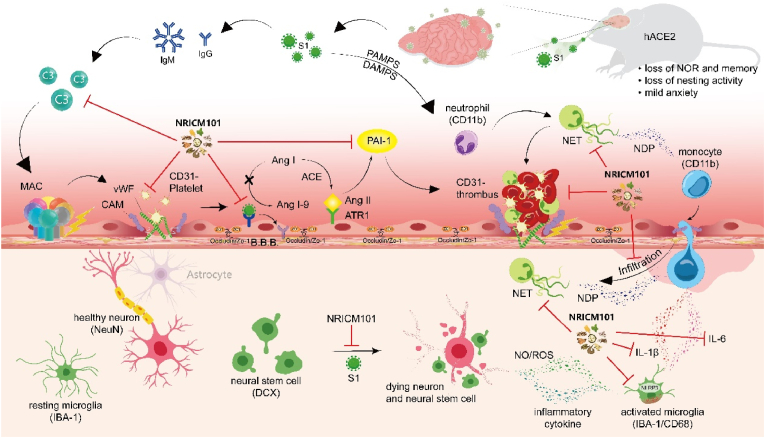
Neurovascular damage and inflammation in COVID-19 associated brain fog follow this sequence: (1) S1 protein activates the complement pathway (C3), leading to membrane attack complex (MAC) formation and endothelial cell impairment; (2) endothelial cell activation increases CD31 (PECAM-1) and vWF expression; (3) S1 mediated damage-associated molecular patterns (DAMPs)/pathogen-associated molecular patterns (PAMPs) activate CD11b + immune cells, releasing neutrophil-derived protein (NDP) and forming neutrophil extracellular traps (NET); (4) ACE2 receptor downregulation by S1 protein binding followed by ATR1 activation, in turn, stimulates PAI-1 formation, causing platelet aggregation and microthrombi (CD31-thrombus); (5) Blood-brain barrier (B.B.B.) damage (lose of occludin and ZO-1) occurs, allowing serum protein leakage into the perivascular area and infiltration of the neural tissue by CD11b + immune cells, activating microglia (CD68/IBA1) and inducing NLRP3 and IL-1β expression; (6) These effects result in neuronal (NeuN+) damage and neural stem (DCX+) cell demise, causing COVID-19 brain fog such as loss of NOR memory and nesting activity. NRICM101 appears to interact with multiple critical pathways involved in neurovascular impairment and inflammation, which are associated with COVID-19-induced brain fog. This interaction, primarily initiated by the S1 protein of the virus, suggests a potential therapeutic effect of NRICM101 in mitigating the cognitive symptoms commonly referred to as brain fog.

To gain deeper insights into the *in vivo* effects of NRICM101 (1.0, 1.5, and 3.0 g/kg) on S1-induced brain fog in hACE2 transgenic mice, we conducted a genome-wide transcriptome analysis using RNA-seq and NGS technologies, specifically Illumina sequencing platforms. The objective of this analysis was to identify and characterize the differentially expressed genes (DEGs) induced by S1 protein that could be influenced by the NRICM101 treatment.

To visualize the overall gene expression patterns and identify the relationships between different groups of mice, a principal component analysis (PCA) plot was constructed. The PCA plot effectively captured the differences in gene expression patterns among the various experimental groups. Notably, the gene expression pattern of NRICM-treated mice (S1+NRICM101) exhibited greater proximity to that of the sham mice (Sham), with the NRICM101 (1.5 g/kg) being the most potent one, indicating a tendency towards a more normalized gene expression profile (Fig. 8A). This result suggests that NRICM101 treatment may exert a regulatory influence on the gene expression changes induced by S1, potentially mitigating the brain damaging effects.

A volcano plot was generated to illustrate the significant impact of NRICM101 on S1-induced DEGs. The analysis revealed a total of 701 DEGs that exhibited significant changes in expression levels due to S1 treatment (632 upregulated and 69 downregulated genes, S1+saline compared to the sham) and were further influenced by NRICM101 (1.5 g/kg) (521 upregulated genes and 16 downregulated genes) (Fig. 8B). This extensive set of DEGs provides valuable information about the transcriptional alterations occurring in the context of S1-induced brain fog and the modulatory effects of NRICM101.

According to the disease ontology (DO) gene-concept network analysis, NRICM101 treatment led to significant suppression of genes involved in positive regulation of cytokine production, leukocyte mediated immunity, and positive regulation of defense response (Fig. 8C, supplementary). This suggests that NRICM101 may counteract these detrimental inflammatory pathways, contributing to the preservation of brain damage.

In addition, KEGG pathway enrichment analysis is a widely utilized bioinformatics and genomics research method that allows for the identification of biological pathways that are significantly enriched with DEGs or other gene sets. According to KEGG analysis, S1 treatment resulted in significant enrichment of genes involved in complement and coagulation cascades (Fig. 8D, supplementary). This suggests that S1 may modulate cellular processes related to activation of the complement system, finally leading to B.B.B. damage; however, this pathway was no longer activated in the NRICM101 treatment group. Some NGS results were adopted in Table 1. Our findings from gene profiling were consistent with the results obtained from image-based data analysis (Fig. 7).

**Table 1:** Gene expression profile adopted from NGS.

Symbol	Log2 Fold Change	
	S1 vs Sham	S1+101 vs S1
Il-6	2.76	−3.42
Il-1β	2.34	−2.30
CD68	3.58	−3.81
IBA-1	3.95	−1.67
vWF	4.46	−2.48
C3	2.97	−3.26
NLRP3	6.05	−2.48
DCX	−1.73	2.86
Wnt6	0.43	1.27
NeuN	−1.05	1.91
Occudin	−1.32	2.64

Gene expression profiling was performed using NGS (next-generation sequencing). The experimental protocol was designed and performed on hACE2 mice with SARS-Co V-2-S1 protein (400 μg/kg)-induced brain fog divided into 4 subgroups, including (1) a saline control (Sham) group, (2) an S1 plus saline-treated (S1) group, and (3) an S1 plus NRICM101 (1.5 g/kg, p.o.)-treated (S1 + 101) group.

## 4 Discussion

Research into long COVID has documented its multisystem manifestations, which encompass various physical and neuropsychiatric symptoms that persist after infection.^32^, ^33^, ^34^, ^35^ These lingering symptoms can take a substantial toll on the quality of life experienced by individuals who have recovered from COVID-19.^36^, ^37^, ^38^ The findings presented in this study shed light on the crucial issue of post-COVID-19 complications, particularly persistent symptoms such as cognitive difficulties and brain-related impacts. These symptoms have been linked to inflammation and cognitive impairment, leading to a pressing need for effective treatments. The study highlights NRICM101 as a promising candidate for addressing COVID brain fog. The observation that over 2.0 million patients globally received NRICM101 for COVID-19 and experienced reduced rates of intubation and hospitalization^14^ is a noteworthy point, suggesting the potential benefits of this treatment in managing severe outcomes.

While it has been previously reported that SARS-CoV-2 infection induced microglial cell activation and neuronal loss, there is a notable absence of brain fog behavior data that directly correlate with those findings.^10^^,^^11^ In this study, we present a pioneering correlation between SARS-CoV-2 spike protein S1 administration-induced brain fog behavior and the robust complement activation and inflammation affecting the microvascular architecture of the B.B.B. This cascade of events results in the impairment of neurons and neural stem cells within the cortex and hippocampus, ultimately culminating in brain fog.

However, employing a moderately mild respiratory model of COVID-19 in animals, those researchers illustrated that introducing SARS-CoV-2 to mice triggered comparable patterns of reactive microglia primarily affecting white matter, loss of oligodendrocytes, hindered neurogenesis, and heightened levels of CCL11 at the initial stage. Despite these findings, no cognitive decline behaviors were detected. These factors could potentially contribute to cognitive decline following mild COVID-19.^10^^,^^11^ In contrast, utilizing a relatively severe COVID-19 animal model (resulting in fatalities and significant weight loss) in this study, we identified that it was IL6 and IL-1β, rather than CCL11 (also known as eotaxin-1), that played a pivotal role in mediating the inflammatory responses induced by SARS-CoV-2 spike protein S1 that led to brain fog. The difference between our findings and those of other models lies in the severity of the COVID-19 impact (severe versus mild). The severity of brain fog varied based on the delivery method of the SARS-CoV-2 spike protein S1 in mice. Severe COVID-19, induced by microinjecting the S1 protein into the internal carotid artery, led to death and significant weight loss due to extensive inflammation and systemic effects impacting the brain. Conversely, mild respiratory COVID-19, induced by intratracheal infection with AAV-hACE2 and a bolus injection of 10^11^ genome copies of AAV-hACE2 into the trachea, did not cause death or significant weight loss. This mild model showed no brain fog behavior, no death of mature neurons (NeuN + cells) in the brain cortex or hippocampus, and no significant neurological deficits.

These models address different clinical questions and severities: systemic influence (transgenic mice) and local influence (trachea infection) on hACE2 activation.

A recent study has established a connection between two biomarker profiles, namely, specifically elevated levels of fibrinogen and elevated D-dimer, as determined through routine blood tests. The association of these biomarkers with cognitive impairment is potentially indicative of COVID-19-related coagulation abnormalities involving thrombosis in the cerebral or pulmonary blood vessels. Nevertheless, it should be noted that the proposed mechanisms remain speculative, and further research is required to more comprehensively investigate these findings.^39^

The link between S1 protein administration, inflammation, microglia activation, cytokine production, and various markers of immune response is compelling. The subsequent damage to neurovasculature and disruption of the B.B.B. underscores the severity of these changes. Comparable results have shown that COVID-induced antibody-mediated complement activation directed against the endothelial cells is the most likely initiating event that leads to neurovascular leakage, platelet aggregation, neuroinflammation, and neuronal injury.^40^ Lee's report linked COVID-19 to vascular damage alongside heightened endothelial cell activity. Platelet clusters and microthrombi were noted along vessel walls, resulting from activation of the classical complement pathway that formed immune complexes on endothelial cells and platelets. In surrounding vessels, immune cell infiltration consisted mainly of macrophages and some CD8^+^ T cells, with occasional CD4^+^ T cells and CD20^+^ B cells detected.^40^ However, our study revealed that the formation of NET by reducing ACE2 expression through S1 binding^13^^,^^20^^,^^22^ was linked to the infiltration of CD11b^+^ immune cells in combination with the activation of platelets, shedding light on another crucial role in our animal model of cognitive impairment.

SARS-CoV-2 spike protein S1 administration causes ACE2 downregulation, leading to microvascular thrombosis through increased angiotensin II expression and elevated PAI-1 levels.^22^ Hospitalized COVID-19 patients also exhibit heightened fibrinogen and vWF levels, which further promote microvascular thrombosis. Additionally, SARS-CoV-2 activates the NOD-like receptor protein 3 (NLRP3) inflammasome, inducing IL-1 family production within microglia. This process enhances tau hyperphosphorylation and aggregation, and it potentially increases the risk of Alzheimer's disease.^41^

The current animal study offers a promising viewpoint, highlighting the efficacy of NRICM101 when administered at clinically relevant doses. This compound demonstrates its effectiveness in mitigating the observed pathological changes through multiple targeted mechanisms. Given the complexities of post-COVID pathology, especially concerning persistent symptoms such as cognitive compromise and brain-related effects, the choice of NRICM101 for treating COVID-related brain fog is both reasonable and compelling. This research highlights its efficacy in an animal model by addressing key factors such as microglia activation, cytokine production, and inflammation, as well as complement and NET activation. NRICM101 appears to play a role in the preservation of neurovascular integrity, conservation of neurons and neural stem cells, and restoration of B.B.B. function.

Nevertheless, the active components in this formulation (NRICM101) and their mechanisms in aiding disease recovery require further elucidation. Key insights from our prior research centered on the inhibition of the 3CL protease, the interplay between hACE2 and the spike protein, and the initiation of the cytokine storm, all of which are vital to inhibition of the virus's detrimental effects by NRICM101. In our research,^13^ we identified 17 primary constituents of NRICM101 that could provide therapeutic advantages. Particularly, baicalin and its derivative, baicalein, stood out as central figures. The researchers posit that the efficacy of NRICM101 stems from the synergistic effects of its components, with baicalin and baicalein being central to its therapeutic properties.^13^ NRICM101 received official drug certification on May 9, 2024, for treating external infectious diseases and epidemics, and for effectively relieving lung symptoms, clearing heat, detoxifying, widening the chest, and reducing phlegm. (https://service.mohw.gov.tw/DOCMAP/CusSite/TCMLResultDetail.aspx?LICEWORDID=01&LICENUM=061606).

Furthermore, animal testing of NRICM101 for toxicity is crucial. The Food Industry Research and Development Institute, a renowned nonprofit organization in Taiwan known for its expertise in animal toxicity studies, has conducted thorough safety and genotoxicity evaluations on NRICM101 (Taiwan Qingguan No.1). Their analysis provided the following succinct findings: A subacute toxicity test, a single-dose oral acute toxicity test, and genotoxicity evaluations showed that, at the specific doses of 1.6, 3.2, and 4.8 g/kg body weight, NRICM101 has a robust safety record in both rats and mice without any signs of genotoxicity (https://www.nricm.edu.tw/p/406-1000-6855,r61.php?Lang=zh-tw). However, considering the long-term use (over six months) of NRICM101 for treating post-COVID symptoms such as brain fog and fatigue, it is crucial to conduct long-term toxicity studies in animals in the future.

Animal models are pivotal for acute COVID-19 research and treatment evaluations, but they do not fully replicate post-COVID human conditions. To understand post-COVID-19 neurological effects, a blend of various models and specific experiments might be essential.^42^ It would be worthwhile to consider the appropriateness of using hACE2 mice with S1 protein administration to simulate COVID-19 brain fog when studying the effects of NRICM101. Fontes-Dantas' study found no weight loss from brain spike protein infusion,^43^ but they used normal Swiss mice, unlike our hACE2 transgenic mice.^20^^,^^22^ This receptor difference could account for the varied results. Translating these observations to humans will demand thorough evaluation and possibly more clinical studies. Further exploration of NRICM101's mechanisms of action, potential side effects, and applicability to human patients is warranted. Additionally, clinical trials involving human subjects could provide more comprehensive insights into its efficacy and safety in addressing COVID-19 brain fog. Moreover, fatigue is a well-documented phenomenon in neuroinflammatory conditions.^44^ A previous study has indicated the existence of a synergistic relationship between brain fog and fatigue, meaning that increased fatigue tends to worsen the experience of brain fog.^45^^,^^46^ To better understand the underlying mechanisms of fatigue and neurocognitive impairment in the context of COVID-19-related brain fog, further research is warranted.

Our findings reveal that the neurovascular damage and inflammation in COVID-19 brain fog involve the following sequence: (1) S1 protein activates the complement pathway, forming MAC and impairing endothelial cells; (2) endothelial cells increase CD31 and vWF expression; (3) S1-mediated DAMPs/PAMPs activate CD11b^+^ immune cells, releasing NDP and forming NET; (4) S1 protein binding downregulates ACE2, activating ATR1 and stimulating PAI-1 formation, leading to platelet aggregation and microthrombi; (5) B.B.B. damage occurs, allowing serum protein leakage and the infiltration of neural tissue by CD11b^+^ immune cells, activating microglia and inducing NLRP3 and IL-1β expression; (6) neuronal and neural stem cell damage results in cognitive symptoms like memory loss. NRICM101 interacts with pathways involved in neurovascular impairment and inflammation, suggesting it may mitigate brain fog symptoms by addressing the effects of the S1 protein (Fig. 9, black arrow line). However, treating S1-administered mice with NRICM101 reduced neuroinflammation and neural cell loss while alleviating declines in cognitive function and nesting abilities (Fig. 9, red line with inhibitory symbol). Our study emphasizes the protective potential of NRICM101 against brain fog in COVID-19 patients. Further clinical trials are required to validate our findings and to ensure the safety and effectiveness of NRICM101 in treating COVID-19-induced brain fog. This research holds significance, as it potentially introduces a new therapeutic approach to alleviate the long-term neurological impact of COVID-19.

## 5 Conclusion

This study offers a promising approach to addressing the cognitive challenges of COVID-19 brain fog. The ability of NRICM101 to reduce S1 protein-induced inflammation and complement and NET activation could lead to new treatments, improving the quality of life for those affected by this condition.

## Declaration of competing interest

The authors declare no conflict of interests.
