# Supplementary material for: NRICM101 in combatting COVID-19 induced brain fog: Neuroprotective effects and neurovascular integrity preservation in hACE2 mice
Source: J Tradit Complement Med. 2024 Jul 3;15(1):36–50. doi: 10.1016/j.jtcme.2024.07.001 (PMC11725119; doi:10.1016/j.jtcme.2024.07.001)
Supplement: Multimedia component 1 [file mmc1.docx]

**Supplementary data**

**Fig. 1**


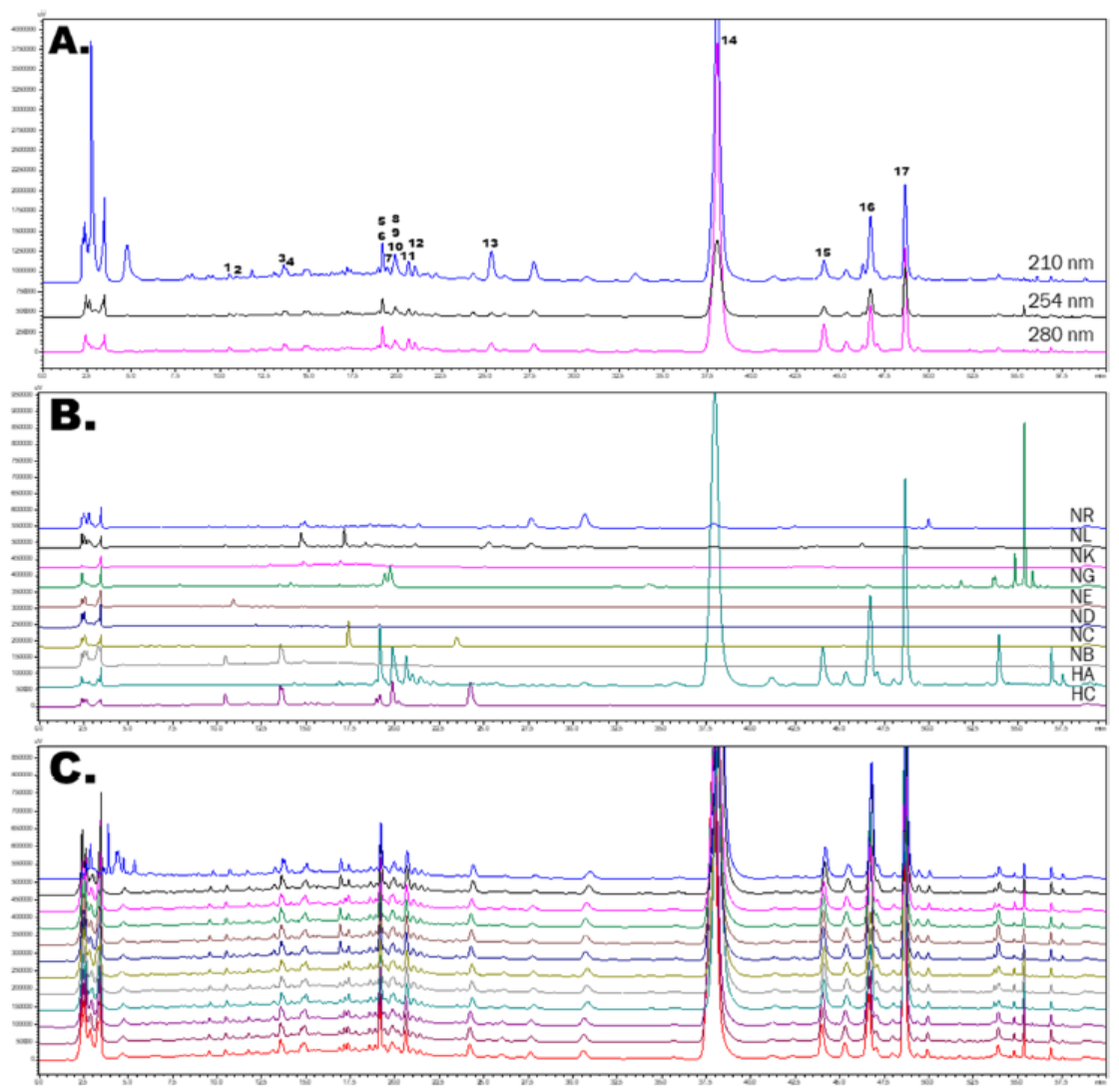


The HPLC fingerprint profiles of NRICM101 decoction, 10 single herbs, and 12 batches of NRICM101. (**A**). The HPLC profiles of NRICM101 decoction at 210, 254, 280 nm. **1**: 3-*O*-Caffeoylquinic acid; **2**: Epigoitrin; **3**: 5-*O*-Caffeoylquinic acid; **4**: 4-*O*-caffeoylquinic acid; **5**: Rutin; **6**: Chrysin 6-*C*-arabinoside-8-*C*-glucoside; **7**: Liquiritin; **8**: Acetoside; **9**: Quercetin 3-galactoside; **10**: Quercetin 3-glucoside; **11**: Chrysin 6-*C*-glucoside-8-*C*arabinoside; **12**: Scutellarin; **13**: Quercetin 3-rhamnoside;**14**: Baicalin; **15**: Norwogonin 7-*O*-glucuronide; **16**: Oroxyloside; **17**: Wogonoside. (**B**). The HPLC fingerprint of the 10 single herbs at 280 nm. HA: Scutellaria root (*Scutellaria baicalensis*); HC: Heartleaf Houttuynia (*Houttuynia cordata*); NB: Mulberry Leaf (*Morus alba*), NC: Saposhnikovia Root (*Saposhnikovia divaricata*); ND: Mongolian Snakegourd Fruit (*Trichosanthes kirilowii*); NE: Indigowoad Root (*Isatis indigotica*); NG: honey-fired Liquorice Root (*Glycyrrhiza glabra*); NK: Magnolia Bark (Magnolia officinalis); NL: Peppermint Herb (*Mentha haplocalyx*); NR: Fineleaf Schizonepeta Spike (*Schizonepeta tenuifolia*). (**C**). The HPLC fingerprints of 12 batches of decoction obtained from the TCM pharmacies of two medical centers at 280 nm.

**Fig. 5A**


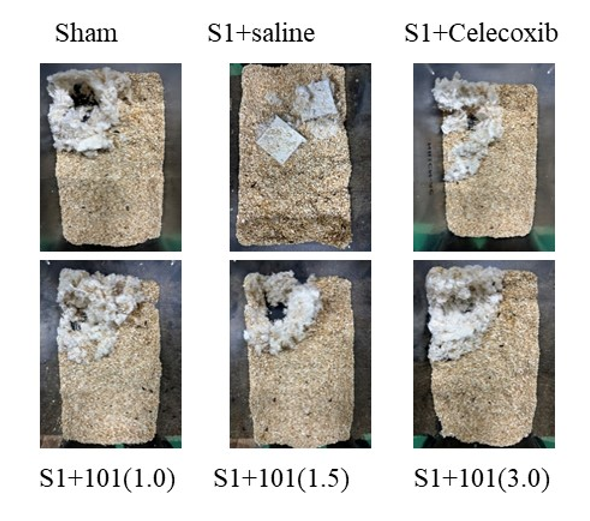


**Fig. 8C**

**
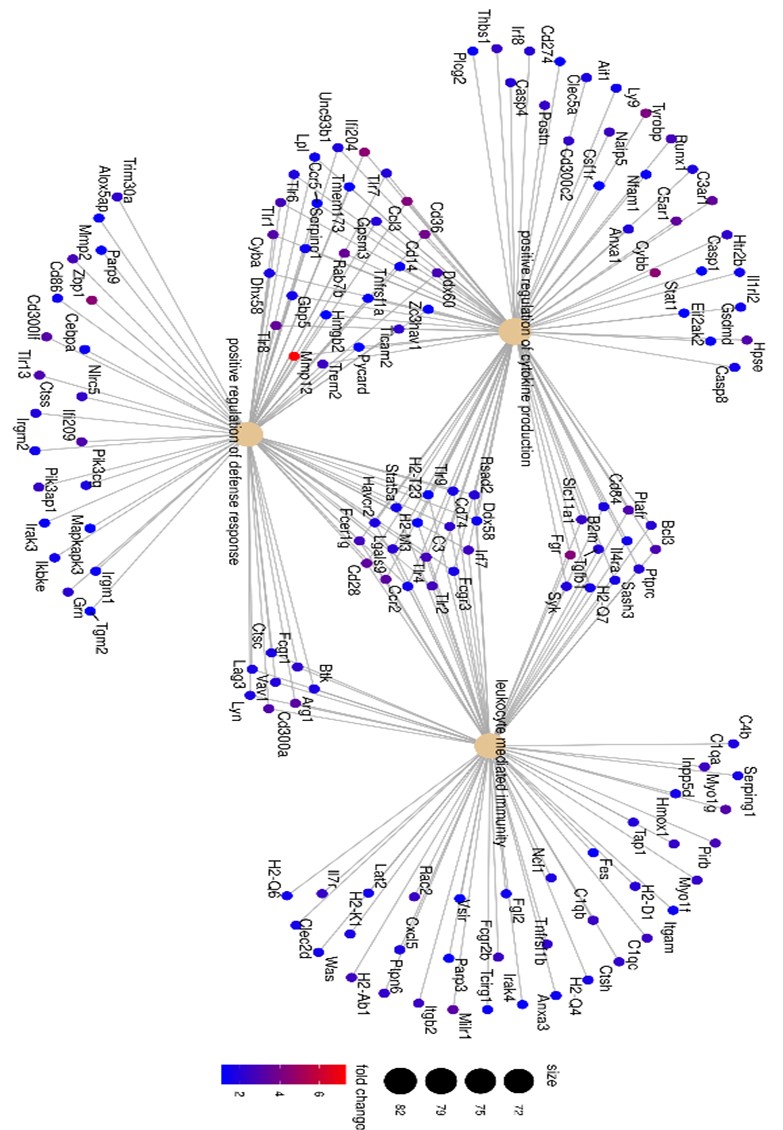
**

**Fig. 8D**

**
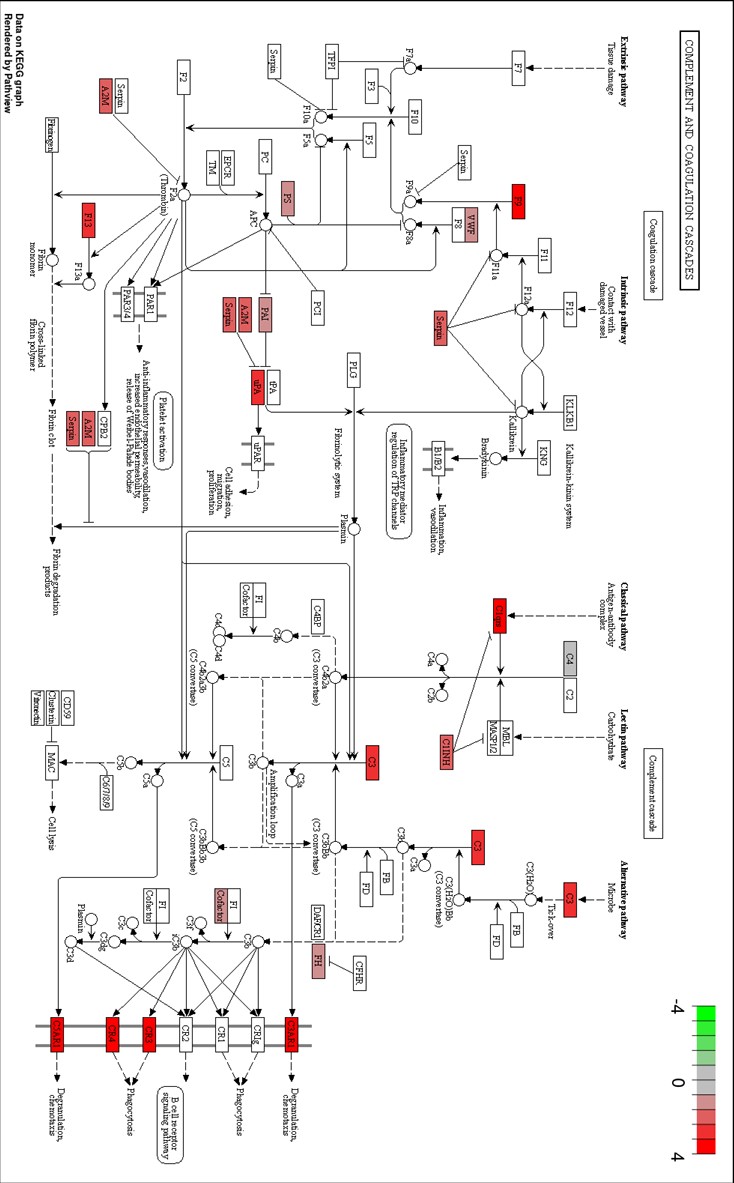
**
